# Thirty-day Emergency Department Utilization after Distal Radius Fracture Treatment: Identifying Predictors and Variation

**DOI:** 10.1097/GOX.0000000000002416

**Published:** 2019-09-10

**Authors:** Jacob S. Nasser, Ching-Han Chou, Kevin C. Chung

**Affiliations:** From the * Section of Plastic Surgery, Department of Surgery, University of Michigan Medical School, Ann Arbor, Mich.; † Center for Artificial Intelligence in Medicine, Chang-Gung Memorial Hospital, Taoyuan, Taiwan; ‡ Section of Plastic Surgery, Professor of Surgery, Assistant Dean for Faculty Affairs, University of Michigan Medical School, Ann Arbor, Mich.

## Abstract

Unplanned hospital visits are costly and may indicate reduced care quality. In this analysis, we aim to investigate the emergency department (ED) utilization for patients 30 days after treatment for a distal radius fracture (DRF) with an emphasis on DRF-related diagnoses of complications and examine nationwide variation in returns to the ED after treatment.

**Methods:**

In this population-based analysis, we used the Truven MarketScan database to identify adult patients who underwent treatment for their DRF. We examined primary diagnoses upon ED visits to assess for relation to treatment. Multivariable logistic regression was performed to evaluate the risk of DRF-related ED visits and pain-related diagnoses.

**Results:**

Of the patients who underwent treatment for a DRF, 2% of surgical patients and 4% of nonsurgical patients visited the ED within 30 days after treatment with a DRF-related diagnosis. The most common diagnoses for all patients were pain-related diagnoses. Patients receiving internal fixation were less likely to experience a DRF-related visit compared with patients receiving other surgical treatments most likely because of no exposed hardware. In addition, patients receiving surgical in the South were more likely to visit the ED with a pain-related diagnosis compared with any other region.

**Conclusions:**

Policy makers should include ED visits after treatment for outpatient hand conditions as indicators of care quality. Given the geographic differences in the ED utilization for this patient population, collaborative initiatives and institutional-level policy may help reduce variation in care for patients after treatment for their fracture care.

## Abstract 2

Supplemental Digital Content is available in the text.

## INTRODUCTION

Unplanned hospital visits have been identified as a substantial financial burden on the healthcare system. Researchers estimated that 20% of United States Medicare beneficiaries experience unplanned readmission to the hospital following discharge, costing approximately 17 billion dollars annually.^1,2^ There have been efforts made by various organizations to reduce spending in healthcare and promote high-quality care.^3–5^ Despite unplanned readmissions being used as an indicator of healthcare quality, emergency department (ED) visits after a common outpatient procedure are rarely used.^4,5^

A substantial portion of patients seek care at the ED because of preventable conditions, poor care management, or inadequate access to care. Estimates suggest that 14%–27% of ED visits could be treated in a different setting.^6^ Therefore, researchers have investigated the utilization of the ED for patients undergoing common surgical procedures such as mastectomy, breast reconstruction, major joint replacement, and thoracic surgery to identify patients with an increased risk of returning to the ED after a surgery.^7–10^ However, these studies fail to assess the diagnoses upon ED visits for potential relation to the condition or procedure of interest.

Distal radius fractures (DRFs) are one of the most common musculoskeletal injuries.^11^ As the number of individuals experiencing DRFs increases,^12^ identifying ways to improve the quality of care and reduce cost becomes more imperative. Curtin and Hernandez-Boussard^13^ found that 9% of patients receiving surgical treatment for a DRF were readmitted within 30 days of surgery. Although the authors used data from different states, the data were not nationally representative. In addition, the diagnoses upon return to acute care were not assessed for potential relation to a DRF. Given the efforts to provide high-value healthcare at a low cost, identifying particular patients with a greater risk of unplanned hospital visits may drive policy aimed at including various types returns to acute care in quality metrics used by organizations focused on improving quality.

The purpose of this population-based study is to examine the DRF-related ED utilization for patients within 30 days of treatment for a DRF. We define a DRF-related diagnosis as a primary diagnosis for a condition likely to result from the DRF itself, treatment for a DRF, or poor medical care. Our specific objectives are to (1) identify the rate of the DRF-related ED utilization within 30 days of treatment, (2) examine the diagnoses upon a DRF-related ED visit, and (3) determine factors associated with increased risk of returning to the ED with a pain-related diagnosis.

## METHODS

### Data Source

We conducted an analysis of the Truven MarketScan Commercial Claims and Encounters and Medicare Supplement and Coordination of Benefits (MarketScan) database from 2009 to 2016. The MarketScan dataset contains one of the largest collections of deidentified patient information used for healthcare research with data from over 32 billion service records. Each patient is identified through a unique identifier code, which permits researchers to longitudinally track the patient throughout the duration of care for the specified medical condition. Our study was considered exempt by our institution’s institutional review board because the database contains publically available, deidentified data.

### Case Selection

We identified patients using an algorithm of International Classification of Diseases, Ninth and Tenth Revision, Clinical Modification codes (ICD-9, ICD-10) and Current Procedural Terminology codes (**see appendix, Supplemental Digital Content 1**, which displays codes used in analysis, http://links.lww.com/PRSGO/B193).

**Fig. 1.**
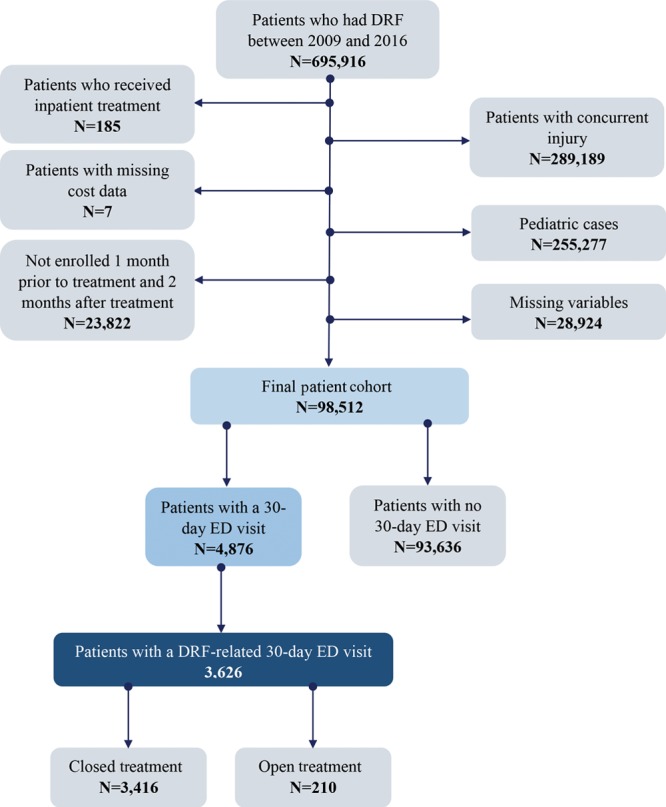
Flowchart of patient cohort.

The full exclusion and inclusion criteria used to obtain the final patient cohort are illustrated in Figure 1. We included claims for adult patients who received outpatient treatment for their DRF to limit heterogeneity. Furthermore, we split the patient sample into 2 subcohorts (surgical versus nonsurgical treatment) to account for differences between surgical and nonsurgical cases. All patients with a concurrent injury on the same day or within 30 days before the DRF were excluded to eliminate potential 30-day ED visits unrelated to the DRF. For example, patients with concurrent hip fractures, lower extremity fractures, or patients with neurological trauma, such as a subdural hematoma, were excluded from our final cohort. We excluded all patients without enrollment in the MarketScan database 1 month before treatment to assess for comorbidities and a preoperative diagnosis of the respective DRF. Furthermore, we excluded patients who were not enrolled 2 months after treatment to ensure all 30-day ED visits were captured in the analysis.

### Assessing for DRF-related Diagnoses

The research team examined each individual primary diagnosis, defined by ICD-9 or ICD-10 codes, for all patients returning to the ED to identify the DRF-related diagnoses. We used the existing literature on DRFs and the clinical experience of the senior members of our research team to stratify the diagnoses.^14–17^ Patients with primary diagnosis codes for obviously unrelated conditions, such as ventricular premature depolarization, were not considered as having a DRF-related ED visit. We considered any codes that may be representative of poor medical care as DRF related. The codes for potentially adverse events have been previously identified by a national expert panel, set up by members of the Utah/Missouri Patient Safety Project.^18–21^ Examples include codes for conditions such as phlebitis and thrombophlebitis, contamination during medical care, among others.^19^

### Variables

Our primary outcome of interest was a DRF-related ED visit within 30 days of treatment. We focused on ED visits with and without subsequent hospital readmissions to examine the ED utilization for patients returning for less severe conditions. Moreover, we examined DRF-related ED visits resulting in readmission by assessing claims that involved an inpatient hospital admission on the same day of ED visit or the next day. We further required a length of stay >0 days for each readmission captured. ED visits within 30 days of treatment were examined because it is the time period used by the Centers for Medicare and Medicaid Services to penalize hospitals with a higher-than-average readmission rate.^22^ In addition, the 30-day metric is commonly used in the literature to examine unplanned readmission rates and ED utilization after a procedure.^7,8,22^

The predictor variables in this analysis included patient demographics and clinical characteristics. Patient demographic data included age, sex, insurance plan, and geographic region (US Census Bureau). Clinical characteristics included treatment modality and Elixhauser comorbidity index. We examined the cost of 30-day ED visits after treatment for a DRF. Cost was determined by summating the values for the following variables in the database: deductible, copayment, coinsurance, coordination of benefits, and payments received by the provider. When calculating the cost, we included all 30-day DRF-related ED visits.

### Assessing for Pain-related Diagnoses

We categorized the DRF-related diagnoses, based on ICD-9 and ICD-10 codes, upon 30-day ED visits to assess for pain diagnoses to the ED. The majority of the pain codes included postoperative pain, pain in the forearm, among similar codes for the pain of the upper extremity.

### Statistical Analysis

Patient and clinical characteristics were examined using chi-square tests for categorical variables and Kruskal Wallis tests for numeric variables. We used multivariable logistic regression models to identify predictors of a 30-day DRF-related ED visit. In addition, we performed a similar analysis of patients visiting the ED 30 days after treatment with a pain-related diagnosis to identify predictors. In our multivariable logistic regression models, we only analyzed claims for the initial 30-day DRF-related ED visit. The *P* value was set at *P* < 0.05 for all statistical analyses.

## RESULTS

### Basic Patient Characteristics

Our analysis included a total of 98,512 patients who underwent treatment for a DRF: 87,501 (89%) patients received nonsurgical treatment and 11,011 patients received surgical treatment (11%). The most prevalent surgical treatment was internal fixation (86%). Basic patient demographic data are listed in Table 1.

**Table 1.** Basic Patient Demographics

	No DRF-related ED Visits (N = 94,886)	DRF-related ED Visits (N = 3,626)	
	N (%)	N (%)	*P*
ED visits resulting in readmission	—	98 (2.70)	—
Patients with >1 ED visit	—	332 (9.16)	—
Discharge to DRF-related ED visit			
0–7	—	2,896 (79.87)	—
8–14	—	367 (10.12)	—
15+	—	363 (10.01)	—
Treatment			
External fixation	314 (0.32)	9 (0.01)	<0.0001
Percutaneous pinning	1,199 (1.22)	34 (0.03)	—
Internal fixation	9,288 (9.43)	167 (0.17)	—
Closed treatment	84,085 (85.36)	3,416 (3.47)	—
Age			
18–34	16,164 (16.41)	635 (0.64)	0.0135
35–44	10,310 (10.47)	452 (0.46)	—
45–54	17,957 (18.23)	691 (0.70)	—
55–64	29,715 (30.16)	1,070 (1.09)	—
65+	20,740 (21.05)	778 (0.79)	—
Sex			
Female	68,013 (69.04)	2,682 (2.72)	0.0026
Male	26,873 (27.28)	944 (0.96)	—
Insurance plan			
Fee-for-service	82,725 (83.97)	3,078 (3.12)	<0.0001
Managed care	12,161 (12.34)	548 (0.56)	—
Median income			
<40,000	2,866 (2.91)	99 (0.10)	<0.0001
40,000–49,999	27,653 (28.07)	996 (1.01)	—
50,000–59,999	42,811 (43.46)	1,551 (1.57)	—
60.000–69,999	15,535 (15.77)	725 (0.74)	—
70,000+	6,021 (6.11)	255 (0.26)	—
Elixhauser comorbidity			
0	73,132 (74.24)	2,716 (2.76)	0.0057
1–3	7,076 (7.18)	274 (0.28)	—
4–8	10,146 (10.30)	430 (0.44)	—
>8	4,532 (4.60)	206 (0.21)	—
Region			
Northeast	17,644 (17.91)	751 (0.76)	<0.0001
North central	23,751 (24.11)	870 (0.88)	—
South	34,699 (35.22)	1,194 (1.21)	—
West	18,792 (19.08)	811 (0.82)	—

### Characteristics of ED Visits

Of the 95,512 patients who received any type of treatment in our sample, 3,626 (4%) visited the ED 30 days after treatment with a DRF-related diagnosis. We found that <3% of patients with a DRF-related ED visit experienced a readmission to the hospital. When stratifying by treatment type, we found that 2% of patients receiving surgical treatment and 4% of patients receiving nonsurgical treatment experienced a DRF-related ED visit 30 days after treatment. In addition, the mean cost for the DRF-related ED visits was $671 (SD ±$1,950) for the surgical cohort and $861 (SD ±$2,186) for the nonsurgical group. When calculating the mean cost for DRF-related ED visits and resulting readmissions, the cost was $1,340 (SD ±$4,646) for the surgical patients and $1,413 (SD ±$887) for the nonsurgical patients.

### Diagnoses on ED Visits

**Fig. 2.**
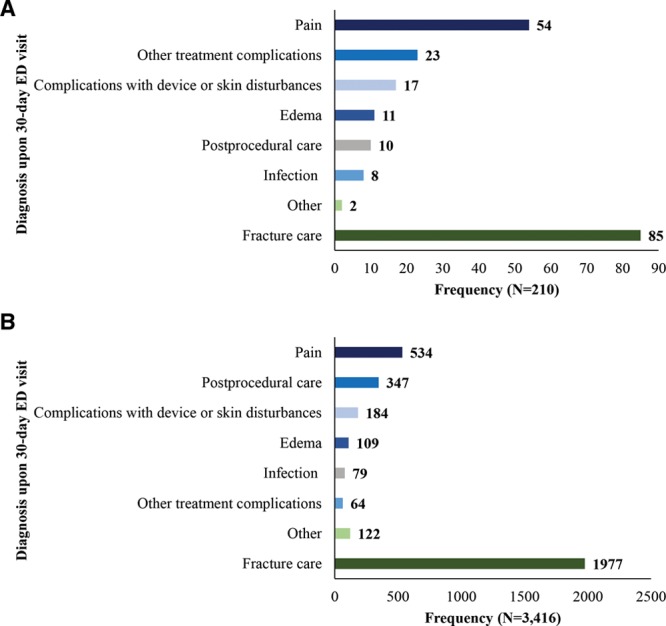
Distribution of Diagnoses Upon Visit to the Emergency Department. A, Distribution of diagnoses upon ED visit following surgical treatment. B, Distribution of diagnoses upon ED visit following conservative treatment. Examples of ICD-9/10 codes included in each category: fracture care (other fractures of distal end of radius), other (poisoning by unspecified drug or medical substance), infection (other postoperative infection, post-traumatic wound infection, not elsewhere classified), postprocedural care (other postsurgical status, other orthopedic aftercare), edema (edema, swelling of limb), complications with device or skin disturbances (mechanical complication due to other implant and internal device, encounter for fitting and adjustment of other specified devices), other treatment complications (postprocedural fever, other specified complications), pain (pain in left wrist, pain in left hand).

The common diagnosis codes for surgical patients experiencing a 30-day DRF-related ED visit, notwithstanding codes for the fracture and miscellaneous reasons, included pain-related diagnoses (26%), other treatment complications (11%), and complications with device or skin disturbances (8%). The distribution of diagnoses upon 30-day ED visits for surgical patients is depicted in Figure 2A. Codes for fracture care include ICD-9/10 codes that are specific to the DRF itself and miscellaneous diagnoses include codes for nausea and other conditions potentially caused by adverse medical care. Among the patients who received nonsurgical treatment and experienced a 30-day DRF-related ED visit, the common reasons for seeking care included a pain-related diagnosis (16%) or postprocedural care (10%). The distribution of diagnoses upon 30-day ED visits for patients receiving closed treatment is depicted in Figure 2B.

### Predictors of DRF-related and Pain-related ED Visits

The odds ratio estimates of factors associated with 30-day DRF-related ED visits after treatment are depicted in Table 2. Surgical patients receiving internal fixation had significantly decreased odds for a DRF-related ED visit [adjusted odds ratio, 0.7 (95% CI, 0.3–1.3)] compared with individuals receiving external fixation or percutaneous pinning. Furthermore, patients with managed care insurance plans were more likely to visit the ED with a DRF-related diagnosis; however, this result was only significant for patients receiving closed treatment. Surgical patients who received internal fixation were less likely to visit the ED with a pain-related diagnosis (Table 3). Nevertheless, this difference was not statistically significant.

**Table 2.** Estimating the Risk of DRF-related 30-day ED Visits

	Surgical Treatment + 30-d DRF-related ED Visit (N = 210)		Nonsurgical Treatment + 30-d DRF-related ED Visit (N = 3,416)	
	Adjusted OR (95% CI)	*P*	Adjusted OR (95% CI)	*P*
Treatment				
Closed treatment	—	—	—	—
External fixation	1.00 (reference)	—	—	—
Percutaneous pinning	1.03 (0.49–2.18)	0.3228	—	—
Internal fixation	0.66 (0.33–1.30)	0.0342	—	—
Age				
65+	1.00 (reference)	—	1.00 (reference)	—
18–34	1.11 (0.63–1.96)	0.6519	1.18 (1.04–1.32)	0.1867
35–44	1.19 (0.67–2.11)	0.9409	1.32 (1.16–1.50)	0.0001
45–54	1.46 (0.93–2.28)	0.1672	1.11 (1.00–1.24)	0.9051
55–64	1.34 (0.89–2.00)	0.3987	1.01 (0.91–1.12)	0.0013
Sex				
Female	1.00 (reference)	—	1.00 (reference)	—
Male	1.08 (0.78–1.50)	0.6337	0.84 (0.77–0.91)	<0.0001
Insurance plan				
Fee-for-service	1.00 (reference)	—	1.00 (reference)	—
Managed care	1.31 (0.89–1.95)	0.1735	1.17 (1.06–1.29)	0.0017
Median income				
<40,000	1.00 (reference)	—	1.00 (reference)	—
40,000–49,999	0.73 (0.38–1.40)	0.9525	1.04 (0.83–1.30)	0.1720
50,000–59,999	0.57 (0.30–1.09)	0.0644	1.03 (0.83–1.29)	0.0809
60.000–69,999	0.67 (0.31–1.48)	0.6534	1.30 (1.02–1.65)	0.0004
70,000+	0.78 (0.33–1.85)	0.8303	1.15 (0.89–1.49)	0.4319
Elixhauser comorbidity				
0	1.00 (reference)	—	1.00 (reference)	—
1–3	0.85 (0.48–1.51)	0.3441	1.08 (0.945–1.230)	0.3616
4–8	1.62 (1.10–2.39)	0.0170	1.16 (1.037–1.298)	0.5806
>8	0.92 (0.42–1.99)	0.6328	1.31 (1.124–1.525)	0.0134
Region				
Northeast	1.00 (reference)	—	1.00 (reference)	—
North central	1.20 (0.71–2.03)	0.0840	0.99 (0.88–1.12)	0.5419
South	0.89 (0.53–1.47)	0.5381	0.95 (0.84–1.06)	0.0283
West	0.78 (0.46–1.32)	0.1874	1.12 (1.00–1.25)	0.0022

OR, odds ratio.

**Table 3.** Estimating the Risk of 30-day ED Visit with a Pain-related Diagnosis

	Surgical Treatment + 30-d DRF-related ED Visit with Pain (N = 54)		Nonsurgical Treatment + 30-d DRF-related ED Visit with Pain (N = 534)	
	Adjusted OR (95% CI)	*P*	Adjusted OR (95% CI)	*P*
Treatment				
Closed treatment	—	—	—	—
External fixation	1.00 (reference)	—	—	—
Percutaneous pinning	1.00 (0.16–6.41)	0.8415	—	—
Internal fixation	0.78 (0.15–4.15)	0.6298	—	—
Age				
65+	1.00 (reference)	—	1.00 (reference)	—
18–34	1.57 (0.38–6.52)	0.3664	1.05 (0.76–1.45)	0.8623
35–44	0.68 (0.16–2.99)	0.3989	1.21 (0.86–1.69)	0.2721
45–54	1.17 (0.36–3.76)	0.7465	1.08 (0.79–1.45)	0.9352
55–64	0.99 (0.34–2.89)	0.8503	1.02 (0.78–1.34)	0.5912
Sex				
Female	1.00 (reference)	—	1.00 (reference)	—
Male	1.60 (0.72–3.54)	0.2488	0.92 (0.73–1.15)	0.4559
Insurance plan				
Fee-for-service	1.00 (reference)	—	1.00 (reference)	—
Managed care	1.64 (0.64–4.24)	0.3052	1.08 (0.83–1.40)	0.5881
Median income				
<40,000	1.00 (reference)	—	1.00 (reference)	—
40,000–49,999	2.39 (0.43–13.21)	0.8730	0.92 (0.51–1.65)	0.9572
50,000–59,999	2.54 (0.46–14.03)	0.9990	0.91 (0.51–1.62)	0.8709
60.000–69,999	7.28 (0.80–66.52)	0.0724	0.82 (0.43–1.56)	0.3831
70,000+	2.39 (0.24–23.64)	0.9246	0.97 (0.49–1.92)	0.7270
Elixhauser comorbidity				
0	1.00 (reference)	—	1.00 (reference)	—
1–3	0.54 (0.12–2.36)	0.5056	0.90 (0.62–1.30)	0.8347
4–8	0.65 (0.22–1.88)	0.6516	0.83 (0.60–1.14)	0.3825
>8	1.18 (0.20–7.17)	0.5759	0.99 (0.66–1.49)	0.6684
Region				
Northeast	1.00 (reference)	—	1.00 (reference)	—
North central	1.57 (0.28–8.82)	0.2959	1.03 (0.73–1.46)	0.7094
South	5.93 (1.20–29.42)	0.0074	0.98 (0.70–1.37)	0.7956
West	3.54 (0.83–15.10)	0.2639	0.99 (0.72–1.36)	0.8883

OR, odds ratio.

### Geographic Variation

We found regional differences in the number of patients visiting the ED 30 days after treatment with a DRF-related or pain-related diagnosis. Surgical patients receiving treatment in the North Central region were more likely to return to the ED with a DRF-related diagnosis, but this result was not statistically significant (Table 2). For patients receiving closed treatment, after adjusting for covariates, the West had a slightly lower probability of a DRF-related ED visit 30 days after treatment and the South had a slightly higher probability of a DRF-related ED visit 30 days after treatment. In addition, patients receiving surgical treatment in the South were 6 times more likely to visit the ED 30 days after treatment with a pain-related diagnosis compared with other regions in the United States (*P* < 0.05), highlighting the opportunity to promote more uniform care (Table 3).

Unplanned hospital visits are used to identify gaps in quality care and therefore reduce discretionary spending in healthcare.^23–25^ The current initiatives aimed at reducing unplanned visits to the hospital focus on readmission rates. For example, the State Action on Avoidable Rehospitalizations program, funded by the Commonwealth Fund, was one of the first statewide initiatives taken to reduce readmissions by targeting individual providers and communities.^26^ These initiatives focused on enhancing postoperative care, patient education, handoff communication, and early follow-up.^27^ Such strategies can be utilized by institutions to provide patients with more resources to effectively and efficiently address their postoperative complaints. In addition, the Hospital Readmission Reduction Program, developed by the Center for Medicare and Medicaid Services, uses 30-day readmission rate to punish hospitals with a higher-than-average readmission rate.

Noureldin et al^28^ examined the 30-day readmission rates for outpatient hand and elbow surgery and found that out of a sample of 14,106 patients, only 169 (1.2%) were readmitted to the hospital 30 days after discharge. Although researchers have found that the rate of unplanned readmissions after outpatient hand surgery is low, complications after treatment for a hand condition may not be serious enough to require readmission. However, patients still experience costly and potentially preventable complications. Thus for some outpatient treatments, for which complications are not as serious, both unplanned readmission and ED visits should be included as a metric of care quality. In our study, we found that ED visits after treatment for a prevalent hand condition occur for both surgical and nonsurgical treatment options. In addition, given the additional, potentially unnecessary costs of a DRF-related ED visit after treatment, policy aimed at reducing ED visits after an outpatient treatment would reduce unnecessary spending during the episode of care. The findings of this analysis highlight the importance of including ED visits following treatment for a common hand condition as a way to measure care quality and decrease wasteful spending.

Complications after surgical treatment for a DRF has been well investigated in the literature.^16,29^ Our research group recently published some findings from the Wrist and Radius Injury Surgical Trial, a randomized clinical trial of surgical treatments for DRF in older patients. We concluded that the complication rate for internal fixation was lower than that of patients receiving casting or an external fixator.^30^ In this population-based analysis of ED visits following a DRF fracture, we found that patients receiving internal fixation had lower odds of a DRF-related ED visit compared with other surgical treatment modalities. In addition, we found that the average cost of 30-day DRF-related ED visits and resulting readmissions is $1,340 for surgical patients. Although various researchers have found that patients electing internal fixation often incur higher costs,^31,32^ the costs of resulting returns to acute care due to potential complications or poor care management should be considered for patient populations with an increased risk of unplanned ED visits and readmissions. Furthermore, future studies may focus on investigating the effect of various clinic factors on the frequency of return visits to the ED, such as early release from the hospital or type of anesthesia.

Researchers found that ED visits after surgery are common and sometimes preventable.^8,13^ However, it is imperative that the diagnoses upon ED visits are assessed for potential relation to the condition of interest. A study by Curtin and Hernandez-Boussard^13^ examined readmission and ED visits after DRF surgery. The researchers found that 9% of patients experienced a readmission to inpatient or emergency care, with a substantial number of patients returning for pain. In our study, we used a nationally representative sample to assess the DRF-related ED utilization for patients after treatment. We found that 2% of surgical patients and 4% of nonsurgical patients returned to the ED 30 days after treatment with a DRF-related diagnosis. In addition, similar to the study by Curtin and Hernandez-Boussard^13^ and other researchers examining the returns to acute care after a procedure,^7,8^ we found a substantial portion of patients visiting the ED return with a potentially preventable pain diagnosis. Methods to reduce postoperative pain may include patient education, nonopioid pain management, care provided through outpatient clinic services, proper counseling on anxiety and fear, and anesthesia-guided protocols anticipating the need for postoperative pain relief.^33–35^ Hospital-level policy focused on patient education may help provide the patients with better guidance regarding care, potentially reducing visits to acute care following treatment. Future prospective studies may study the effect of patient education on reducing the number of unnecessary visits to the ED.

Value-based reimbursement is becoming increasingly popular. Consequently, variation in care has been of great interest for researchers investigating ways to help promote high-quality care.^36,37^ In our study, we found geographic variation in the ED utilization for DRF-related and potentially preventable pain diagnoses 30 days after treatment. Our findings corroborated with other research on variation in ED admission rates across different United States regions.^38^ Given the numerous initiatives to reduce variation in healthcare, these results highlight potential ways to reduce healthcare variation. Researchers have suggested that initiatives to reduce unplanned hospital visits should be community based.^38^ Cultural differences in pain perception and patient education may account for the geographic variation in the ED utilization with pain, representing an avenue to reduce variation and improve care for patients.^39–41^ Future qualitative research investigating patient preferences for pain control after surgery in different regions may shed light on the variation in ED utilization. In addition, national quality collaboratives may reveal provider- and regional-level variation in the management of common hand conditions, opening up an avenue to provide more uniform care to patients.

The implementation of artificial intelligence models could help identify patients with an increased risk of returning to the hospital or the ED after treatment. Clinicians at Massachusetts General Hospital and Harvard Medical School, along with a team from the Massachusetts Institute of Technology, have developed an artificial intelligence model to help surgeons determine the risk for various complications after emergent general surgical procedures. By selecting an outcome of interest, surgeons are then prompted to answer multiple questions about the patients to help the computer predict a particular outcome.^42^ Such technology could be applied to more diverse surgical procedures to predict various outcomes that could result in an unplanned visit to the ED or hospital readmission.

Our study must be interpreted with consideration of its limitations. In this analysis, we only examined ED visits within 30 days of the treatment. Thus, it is possible not all DRF-related ED visits that occurred during the episode of care were not captured in this analysis. Nonetheless, the 30-day metric was used because the time frame is accepted by the Center for Medicare and Medicaid Services to assess unplanned readmission rates. Furthermore, we only used primary diagnoses to examine the cause of the ED visit. Future prospective research studies may be warranted as they will permit more specific assessments of the ED utilization for patients following a specified procedure. In addition, the MarketScan database includes data from individuals with employer insurance. Thus, the dataset contains middle-class patients in the United States with better access to healthcare services compared with patients of a lower socioeconomic status. We postulate that an analysis including both low- and middle-class patients would have a higher rate of ED visits, as many researchers have identified a lower socioeconomic status with a more difficult time finding medical care.^43,44^

As various healthcare systems transition to value-based care, it becomes increasingly important to identify ways to reduce discretionary, wasteful spending. Unplanned ED metrics may provide policy makers a new way to improve the quality of care and reduce the cost for patients receiving outpatient care for common hand conditions. Policy aimed at reducing nationwide variation in the utilization of the ED will further help improve the quality of care for patients after hand surgery. Furthermore, additional efforts by physicians to educate patients on the management of common complications will help reduce the number of unnecessary visits to the ED after surgery.

## ACKNOWLEDGMENTS

We would like to acknowledge the following grants awarded to Dr. Kuo and Dr. Chung for staff salary support specifically for this project: CORPG3G0111, CORPG3G0161, CORPG3H0071, and CIRPG3H0021. Additionally, we would like to thank Lin Zhong for her help with the preliminary statistical analyses.

## Supplementary Material
