# Supplementary material for: Thirty-day Emergency Department Utilization after Distal Radius Fracture Treatment: Identifying Predictors and Variation
Source: Plast Reconstr Surg Glob Open. 2019 Sep 10;7(9):e2416. doi: 10.1097/GOX.0000000000002416 (PMC6799403; doi:10.1097/GOX.0000000000002416)
Supplement: Supplementary file 1 [file gox-7-e2416-s001.pdf]

Supplemental Digital Content 1. Codes Used in Analysis

Category	Code Type	Codes
Distal Radius Fracture	ICD-9	813.40, 813.41, 813.42, 813.44, 813.45
	ICD-10	S52.5, S59.2, S52.6
External Fixation	CPT	20690, 20692
Closed Treatment	CPT	25600, 25605, 29065, 29075, 29085, 29105, 29125, 29126
Percutaneous Pinning	CPT	25606
Internal Fixation	CPT	25607, 25608, 25609
